# Research trends and foci in chondrosarcoma: A bibliometric and network analysis

**DOI:** 10.1097/MD.0000000000040403

**Published:** 2024-11-08

**Authors:** Yuming Yao, Ruhao Zhou, Guang Yang, Bingzhou Ji, Yusheng Li, Jun Zhang

**Affiliations:** a Department of Orthopedics, Xiangya Hospital, Central South University, Changsha, Hunan, China; b Department of Geriatrics, National Clinical Research Center for Geriatric Disorders, Xiangya Hospital, Central South University, Changsha, Hunan, China; c Department of Orthopedics, The Second Hospital of Shanxi Medical University, Shanxi Key laboratory of Bone and Soft Tissue injury repair, Taiyuan, Shanxi, China; d Department of Orthopedics, Changde Hospital, Xiangya School of Medicine, Central South University (The First People’s Hospital of Changde City), Changde, Hunan, China.

**Keywords:** bibliometric analysis, chondrosarcoma, network analysis, Rstudio, VOSviewer

## Abstract

Chondrosarcoma is 1 of the most common malignant bone tumors, with dedicated research being conducted by scientists worldwide. The purpose of this study was to guide researchers in identifying valuable scholars, institutions, and countries, provide recommendations for journal submissions, and explore research trends and hotspots in chondrosarcoma studies through literature analysis. Data for this study were collected from the Web of Science Core Collection website. The R package bibliometrix was utilized for citation metrics analysis, VOSviewer for network analysis, and CiteSpace for generating keywords citation burst maps. The analysis focused on publications from 2000 to 2023, identifying trends, authorship patterns, and collaboration networks. A total of 2085 articles were initially identified, but after excluding non-English articles and those outside the study’s time range, 2022 articles were included. The field comprised 9954 author records, with an average of 6.37 coauthors per document and 13.9% international co-authorships. Publications in chondrosarcoma research have shown an average annual growth rate of 3.9%. The most influential author identified was Tang Chih-Hsin from China Medical University. Significant contributions came from China Medical University and Leiden University, with China showing a dramatic increase in publications while the United States maintained a leading position in the field. The study highlights an increasing trend in chondrosarcoma research publications and identifies key contributors and institutions. Cancer emerged as 1 of the most influential journals in the field. Future research is likely to focus on targeted therapy for refractory chondrosarcomas, indicating a potential new hotspot in the ongoing efforts to understand and treat this malignancy.

## 1. Introduction

Chondrosarcoma is a malignant bone tumor characterized by the production of cartilage stroma by tumor cells.^[1]^ Chondrosarcoma is the most common primary malignant bone tumor after osteosarcoma, accounting for 20% to 27% of primary malignant bone tumors. The peak diagnosis occurs when the patients are in their sixties and seventies.^[2]^ The most common anatomical location is the pelvis, followed by the proximal femur, proximal humerus, distal femur, and ribs.^[3]^ Chondrosarcomas are usually low- or intermediate-grade malignant and are characterized by a slow clinical course and low metastatic potential; however, high-grade malignant chondrosarcomas have a high metastatic potential and poor prognosis, accounting for 5% to 10% of chondrosarcomas.^[4]^ Research is currently focused on novel therapeutic methods for refractory, metastatic, or inoperable chondrosarcomas. Although multiple new treatment options are available for most subtypes of chondrosarcoma, a proportion of subtypes still lack effective treatments other than surgery and remain associated with poor clinical outcomes.^[5]^

Significant advancements in the development and treatment of chondrosarcoma during the past 3 decades have been reported by an increasing number of articles. Over time, bibliometric analysis has become 1 of the effective methods for examining in-depth research trends in a certain field.^[6]^ The evaluation of quantitative and qualitative trends over a certain period can be done using database-based information from bibliometric analysis, which also provides a way to assess the contributions of different countries, journals, and institutes.^[6]^ Thus far, the bibliometric analysis of chondrosarcoma has been rarely reported. Our prior work has demonstrated that bibliometric analysis is quite effective for exploring essential learning fields and pertinent representative literature. It can also be used to validate hotspots.^[7–10]^

In this study, we investigated the publishing trend and research hotspots of chondrosarcoma by analyzing existing literature; citation metrics were calculated using RStudio, and VOSviewer and CiteSpace were applied to conduct keywords and network analysis, and visualization of the results. Through our analysis, we can help researchers find suitable research partners/institutions/countries, provide advice regarding journal submissions, and help researchers capture research hotspots in this field.

## 2. Methods

### 2.1. Data source and search strategy

**Figure 1.**
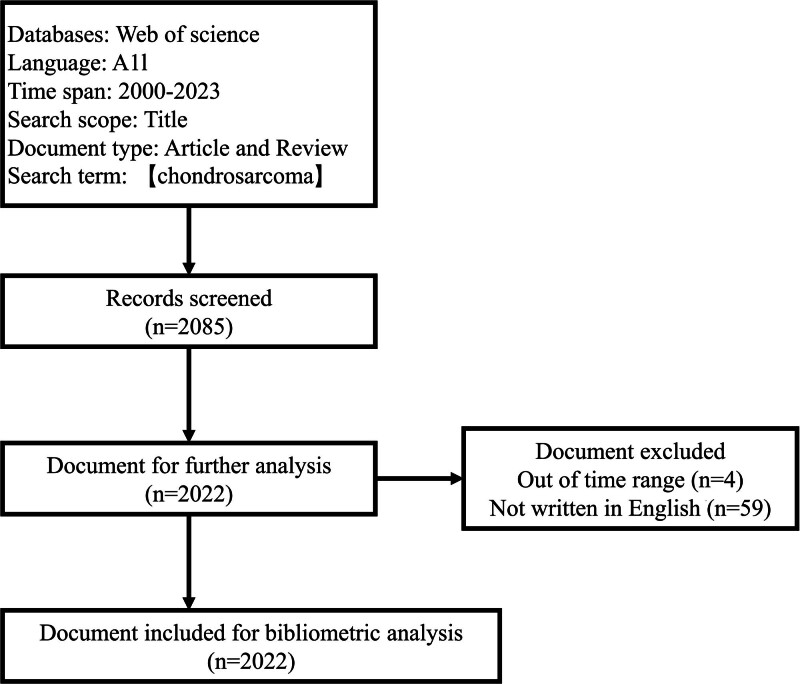
Flow diagram of the search strategy.

Recently, bibliometrics has developed into a characteristic and practical method for statistical analysis of existing literature, and the Science Citation Index Expanded and the Social Science Citation Index from Clarivate Web of Science (WOS) are the most authoritative databases for bibliometric analysis.^[11–13]^ Bibliometric analysis in the chondrosarcoma research field was conducted using the WOS core collections from January 1, 2000, to December 31, 2023. Via a PubMed “Mesh Terms” search, we found that the search term “chondrosarcoma” correlated to all the publications related to the topic. Therefore, conducting a literature search using only this search term ensured that our search results are comprehensive and reliable. The search terms [TI = (chondrosarcoma)] AND [PY = (2000–2023)] AND [DT = (Article OR Review)] (TI: title, PY: publication year, DT: document type) were used to search for articles related to chondrosarcoma research published in any language. The title, which is part of the article, serves as a summary of the other sections and takes on the role of the paper’s cover. We concentrated on the title to obtain a search result that was both accurate and comprehensive. To analyze the recent status of chondrosarcoma research, we restricted the period of publishing from 2000 to 2023. For the data exported from the WOS, the title, document type, and year of publication were checked individually. Data outside the scope of the search terms were excluded. Two researchers used the WOS database to conduct the search in accordance with the search terms, and a third researcher was allowed to participate in the search when the search results diversified. Figure 1 illustrates the search strategy.

### 2.2. Statistical analysis and visualization

In this study, 4 distinct types of software^[7]^ were used: Microsoft Excel 2019 was used to calculate the percentage and frequencies of the published materials; VOSviewer (version 1.6.18) was used to create the bibliometric network analysis; R package bibliometrix^[14]^ was used to calculate the citation metrics.

## 3. Results

### 3.1. Temporal distribution of publications and citations

**Figure 2.**
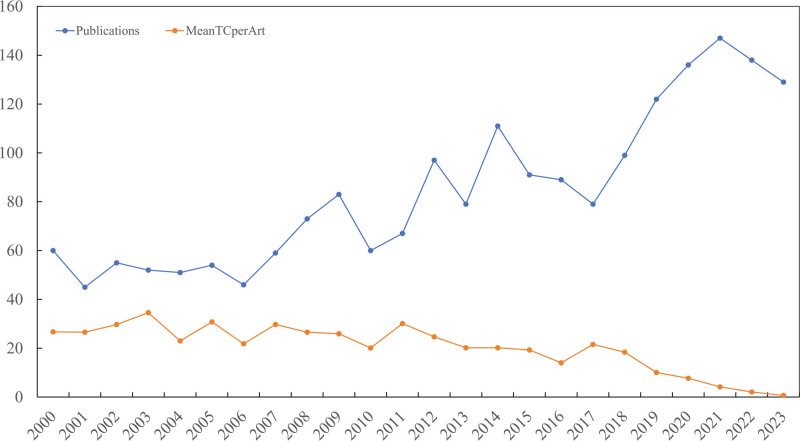
Distribution of publications and citations.

A total of 2085 articles were identified from the WOS database; among them, 59 articles were not written in English, and 4 articles were beyond the time range of our study. Therefore, the remaining 2022 articles were included. Of the included articles, 226 were review-type articles and 1796 were article-type articles. All the papers were published in journal form and no other publication types were observed. Publications increased, with an average growth rate of 3.38% per year from 2000 to 2023. With a total of 45 articles, 2001 was the year with the lowest scientific output. The top 5 years in terms of research output were 2021 (147 articles), 2022 (138 articles), 2020 (136 articles), 2023 (129 articles), and 2014 (111 articles). The average annual article output during 2000 to 2023 was 84 articles. Of these, 14 years did not meet the threshold for scientific output, while the remaining 10 years exceeded this standard. The specific results are shown in Table 1. The highest mean total citation per year was in 2017 (2.69), followed by 2018 (2.63), 2011 (2.15), 2015 (1.93), and 2012 (1.90) (Fig. 2).

**Table 1:** Annual scientific productions and citations.

Year	Publications	MeanTCperArt	MeanTCperYear	CitableYears
2000	60	26.73	1.07	25
2001	45	26.56	1.11	24
2002	55	29.69	1.29	23
2003	52	34.58	1.57	22
2004	51	23.02	1.10	21
2005	54	30.78	1.54	20
2006	46	21.83	1.15	19
2007	59	29.75	1.65	18
2008	73	26.56	1.56	17
2009	83	25.96	1.62	16
2010	60	20.12	1.34	15
2011	67	30.09	2.15	14
2012	97	24.68	1.90	13
2013	79	20.18	1.68	12
2014	111	20.2	1.84	11
2015	91	19.29	1.93	10
2016	89	14.04	1.56	9
2017	79	21.56	2.69	8
2018	99	18.4	2.63	7
2019	122	10.07	1.68	6
2020	136	7.72	1.54	5
2021	147	4.19	1.05	4
2022	138	2.12	0.71	3
2023	129	0.71	0.36	2

MeanTCperArt = mean total citations per article; MeanTCperYear = mean total citations per year.

### 3.2. Prolific authors and co-authorship

**Figure 3.**
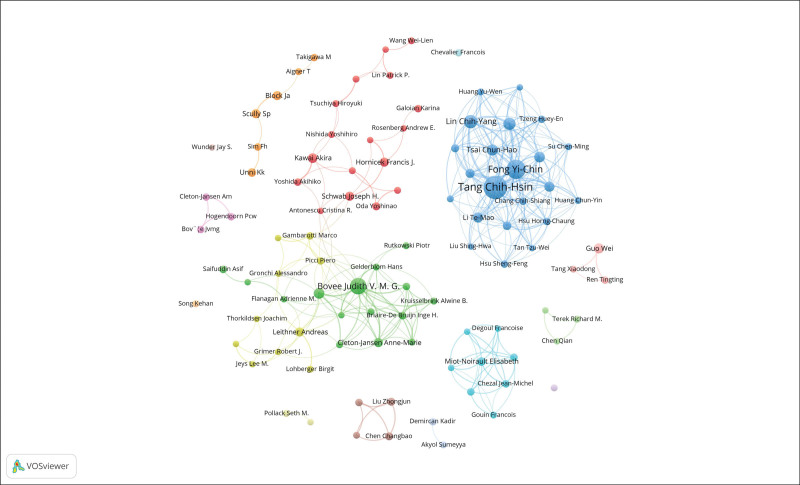
Network and group visualization of co-authorship: Authors in the same color belong to the same group, the more the 2 authors collaborate, the thicker the line between them.

A total of 9954 author records were found in this research field, of which 22 authors independently completed their own articles. coauthors per document was 6.37, international co-authorships were 13.9%. Tables 2 depict the top ten authors distributed by H index. Tang Chih-Hsin (China Medical University, China), with a total of 82 articles and 2591 citations, was the most authoritative author in the chondrosarcoma research field. Fong Yi-Chin (China Medical University, China) with a total of 57 articles and 1818 citations was the second most authoritative author. Two major academic communication groups have formed in the chondrosarcoma research field: research group 1 with Tang Chih-Hsin as the core was the largest, and research group 2 with Bovée Judith V. M. G. as the leader was the second largest. Authors with more than 5 articles are shown in Figure 3. The thickness of the lines between each author is proportional to the number of times they appear together in the same document. The thicker the lines between tags, the more number of times they appear in the same item.

**Table 2:** Top 10 authors distributed by publications.

Rank by H index	Element	Country	Institution	H_index	G_index	M_index	Total citations	Publications	PY_start
1	Tang Chih-Hsin	China	China Medical University	33	45	1.941	2591	82	2008
2	Fong Yi-Chin	China	China Medical University	30	41	1.429	1818	57	2004
3	Bovée J. V. M. G	The Netherlands	Leiden University Medical Center	30	49	1.2	2422	50	2000
4	Hogendoorn Pcw	The Netherlands	Leiden University Medical Center	24	31	0.96	2626	31	2000
5	Lin Chih-Yang	China	Mackay Med Coll	17	24	1.214	616	29	2011
6	Cleton-Jansen Anne-Marie	The Netherlands	Leiden University Medical Center	21	25	0.84	1184	25	2000
7	Wang Shih-Wei	China	Mackay Med Coll	18	23	1.5	765	23	2013
8	Tang Chih-Hsin	China	China Medical University	16	21	1	672	21	2009
9	Kawai Akira	Japan	National Cancer Centre Hospital	11	19	0.478	514	19	2002
10	Yang Wei-Hung	China	China Medical University	15	17	0.833	622	17	2007

### 3.3. Most dominant institutions and countries

**Figure 4.**
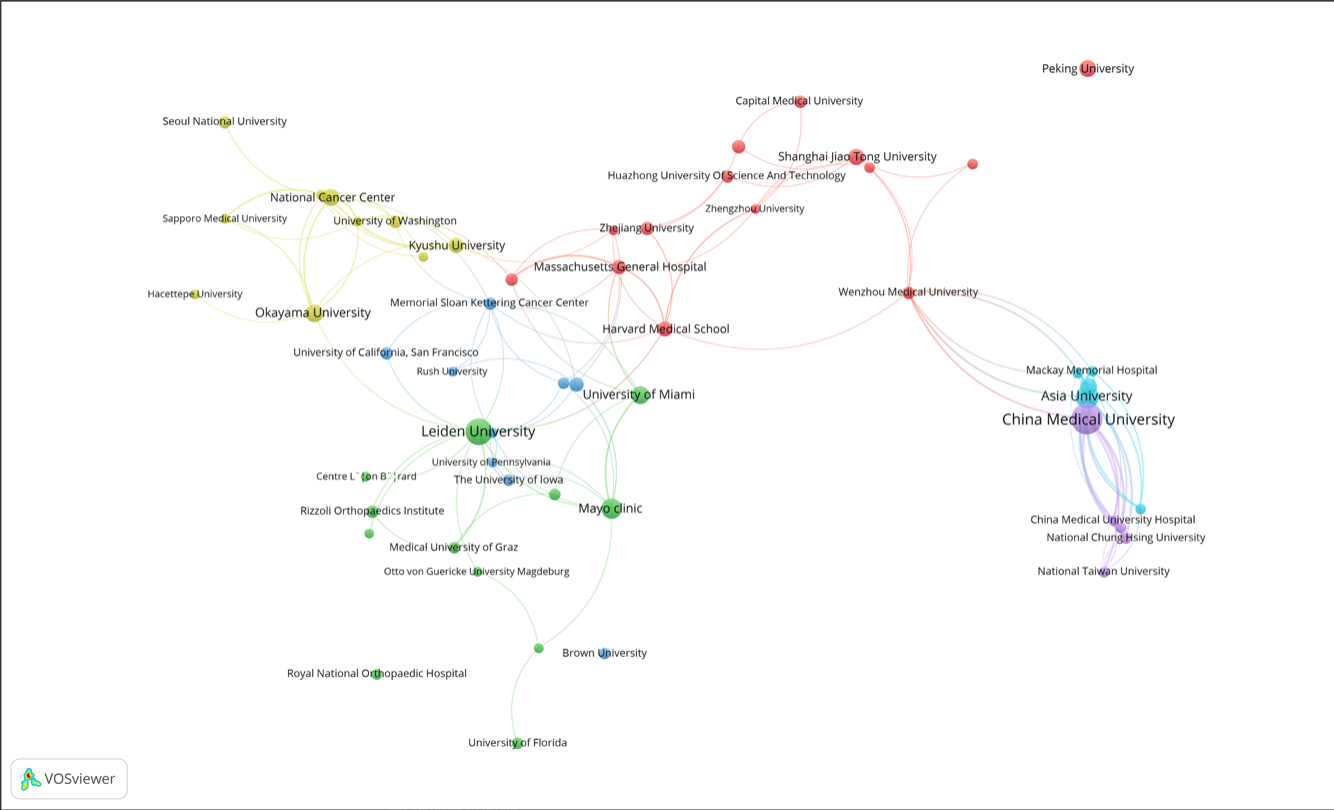
Network visualization of institutions’ collaboration; the more the 2 institutions collaborate, the thicker the line between them. Evolution of contribution of institutions in publication.

A total of 64 countries were included in this study, and the research output was mainly concentrated in the USA, China, Japan, and a few other countries. As indicated in Table 3, the top 10 countries in terms of research output were the USA (434 articles, 10,122 citations), China (408 articles, 382 citations), Japan (207 articles, 2779 citations), Italy (105 articles, 1703 citations), India (80 articles, 216 citations), the UK (74 articles, 2724 citations), Germany (72 articles, 1616 citations), South Korea (66 articles, 854 citations), the Netherlands (66 articles, 2538 citations), France (62 articles, 1020 citations). In contrast to the close research collaboration that Leiden University maintained with other institutions, China Medical University had few research collaborations with other institutions (Fig. 4). Leiden University was a leading institution in this field and was the first authoritative institution to produce influential research work in this area.

**Table 3:** Top 10 Country distributed by publications.

Country	Publications	Freq	Citations	SCP	MCP	MCP_Ratio
USA	434	0.216	10,122	356	78	0.1797
China	408	0.2031	6099	382	26	0.0637
Japan	207	0.103	2779	198	9	0.0435
Italy	105	0.0523	1703	91	14	0.1333
India	80	0.0398	216	76	4	0.0500
United Kingdom	74	0.0368	2724	57	17	0.2297
Germany	72	0.0358	1616	56	16	0.2222
South Korea	66	0.0329	854	58	8	0.1212
Netherlands	66	0.0329	2538	45	21	0.3182
France	62	0.0309	1020	43	19	0.3065

SCP = single-country publications, MCP = multi-country publications.

### 3.4. Preferred journals

**Figure 5.**
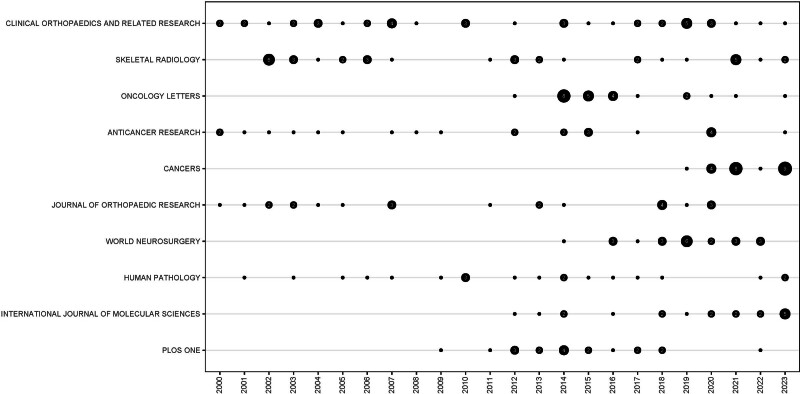
Visualization of publications of the top 10 most productive journals. Publications with less than 2 articles are presented as black dots. Publications with more than 2 articles are presented as a specific number.

The top 10 most productive journals in the chondrosarcoma research field are listed in Table 4. Clinical Orthopaedics and Related Research, with a total of 42 publications, obtained publication rank 1, followed by Skeletal Radiology with a total of 35 articles, while the third was Oncology Letters with 24 articles. 2022 Journal Citation Reports indicated that only Clinical Orthopaedics and Related Research and International Journal of Molecular Sciences were listed in Q1 journals. The number of articles included in cancer journals in this field has gradually increased and gradually occupied a dominant position (Fig. 5).

**Table 4:** Top 10 journals distributed by publications.

Rank	Journal	Publications	% of 2022	IF (JCR 2022)	JIF quartile
1	Clinical Orthopaedics and Related Research	42	2.08%	4.3	Q1
2	Skeletal Radiology	35	1.73%	2.1	Q3
3	Oncology Letters	24	1.19%	2.9	Q3
4	Anticancer Research	23	1.14%	2	Q4
5	Cancers	23	1.14%	5.2	Q2
6	Journal of Orthopaedic Research	23	1.14%	2.8	Q2
7	World Neurosurgery	22	1.09%	2	Q3
8	Human Pathology	20	0.99%	3.3	Q2
9	International Journal of Molecular Sciences	19	0.94%	5.6	Q1
10	PLoS One	19	0.94%	3.7	Q2

### 3.5. Subdisciplines classified by keywords

**Figure 6.**
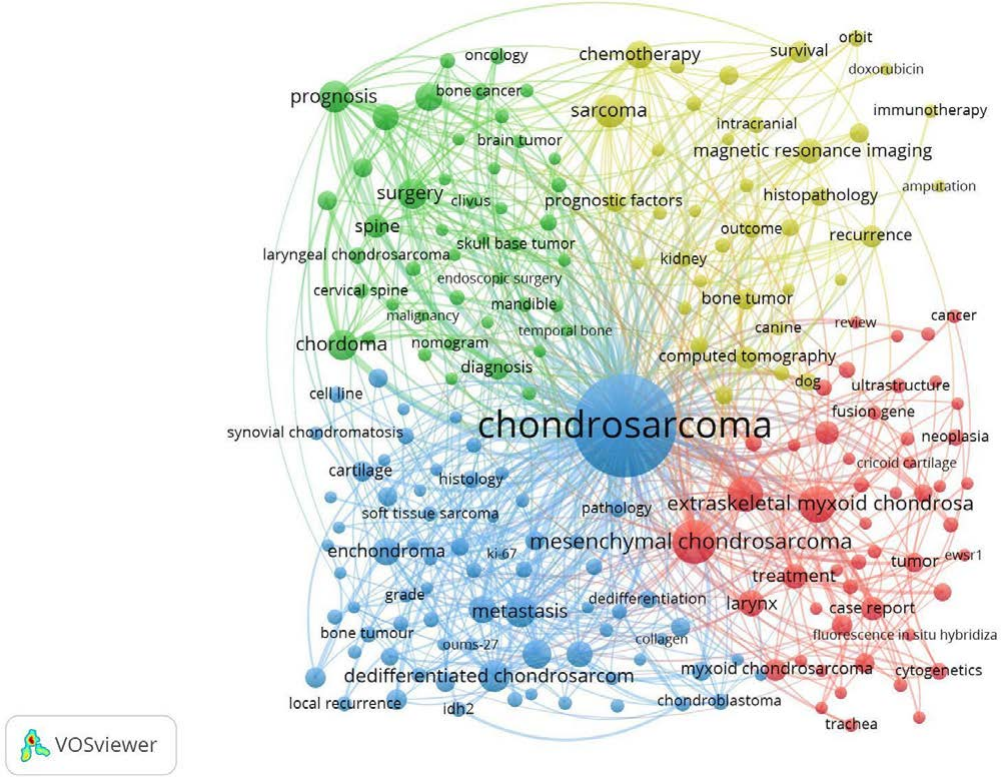
Network visualization map of the author keywords. Keywords in the same cluster are rendered in the same color. The more times 2 keywords appear in the same article, the thicker the line between them. The more times the keywords appear, the bigger their tags.

Mapping author keywords with a minimum of 10 occurrences with the VOSviewer technique revealed that “Chondrosarcoma,” “Bone,” and “Tumors” were the top 3 occurrences of author keywords. As shown in Figure 6, keywords that matched the screening criteria were grouped into 4 clusters. Circles in the same color cluster indicate that the keywords are on the same topic or subfield. Each cluster represents a subfield of chondrosarcoma study. Specifically, as shown in the blue cluster, keywords such as proliferation, angiogenesis, migration, metastasis, apoptosis, IDH1, IDH2, Ki-67, p53, MMP-1, MMP-13, NF-kappaB, SOX9, VEGF, and transcription factors were related to the topic “molecular mechanisms.” Keywords in the green and yellow clusters, such as therapy, surgery, radiotherapy, chemotherapy, doxorubicin, immunotherapy, prognosis, and survival, are related to “treatment and prognosis.” The green cluster is more surgical and the yellow cluster is more medical. Keywords in the red cluster, such as mesenchymal chondrosarcoma and extraskeletal myxoid chondrosarcoma, are related to “chondrosarcoma subtypes.”

## 4. Discussion

Bibliometric analysis is used to quantify the characteristics and scholarly impact of citation classics. Bibliometric and visualized studies can be used to show the current or past status of a specific research field and predict future trends. With a significant increase in the number of articles published over the years, bibliometrics has become the most widely accepted method for assessing the merits of a particular field.^[9,15,16]^ This study analyzed 2022 articles from chondrosarcoma research over the past 3 decades. The number of articles published from 2000 to 2023 showed an upward trend every year and reached a maximum number of 147 in 2021, indicating an increasing momentum in chondrosarcoma research. The downward trend in average citations from 2000 to 2023 suggests that the most recent articles may not be noticed by researchers concerned with chondrosarcomas. However, the average citation level does not fully represent the quality of the articles, as citations from previous publications tend to be higher than those from current ones, and the citation lag contributes to this phenomenon.^[17]^

Four of the top 5 most prolific authors were from China, with another from the Netherlands, reflecting the growing presence of China in this research field. It is worth mentioning that Tang Chih-Hsin and Fong Yi-Chin, 2 of the most prolific authors, are both from China Medical University. China Medical University has therefore made a significant contribution to this field. The number of papers published by a country or institution in a particular field of research is considered an important indicator of the level of their scientific research.^[18]^ Among the institutions, China Medical University, China Medical University Hospital, and Leiden University are the top 3 in the field. China Medical University Hospital and China Medical University can be considered as 1 institution, and the volume of publications is enormous. China Medical University and Leiden University are the 2 institutions that contributed the most to this field. These findings may provide guidance to investigators involved in chondrosarcoma research.

Overall, the United States remains the most productive country in the field of chondrosarcoma research, maintaining an authoritative position. The United States had the most scientific collaborations with other countries such as China, Japan, the United Kingdom, and the Netherlands. China rose to second place in the world in the number of publications and has evolved from an initially marginal country to a valuable country in the field of chondrosarcoma research, which is closely related to the strengthening of China comprehensive national power in recent years, strong investment in scientific research, and gradual improvement of research platforms.^[19]^ However, publications from China are less cited and have received less attention from other countries around the world. One reason may be that newly published articles will be cited less often than older articles, and most publications from China have been published in the last ten years.

Combining the top keywords and related literature, we summarized the 3 research hotspots of chondrosarcoma as follows:

Treatment and prognosis study: the top 10 most frequent keywords “survival,” “prognosis,” and “surgery,” and keywords in the green and yellow cluster (Fig. 6) suggested that treatment and prognosis of chondrosarcoma is 1 of the hotspots. In addition, most of the hot keywords that appeared in recent years were related to treatment and prognosis, including resection, chemotherapy, outcome, surgical treatment, and survival (Fig. 6). The second most cited article in the field of chondrosarcoma is an article by Gelderblom et al on the clinical treatment of chondrosarcoma.^[20]^ The most recent article on the treatment and prognosis of chondrosarcoma also indicates that this area needs further study, especially for unresectable and metastatic chondrosarcoma.^[21,22]^

Molecular mechanisms study: the presence of “p53,” “NF-kappaB,” and “mutation” in the keywords in the blue cluster (Fig. 6) suggested that molecular mechanisms study is a hotspot for chondrosarcoma. In a review, Thoenen et al proposed that the tumor suppressor p53 plays a significant role in the suppression of chondrosarcoma progression, which has advanced the development of new treatment strategies for drug-resistant sarcomas.^[23]^ Experimental studies by Kittiwattanokhun et al observed that chondrosarcoma progression can be inhibited by inhibiting the nuclear factor (NF) signaling pathway.^[24–26]^ Furthermore, the most cited article in the field of chondrosarcoma (Table 2) was from Amary et al on the molecular mechanism of isocitrate dehydrogenase (IDH)1 and IDH2 mutations in chondrosarcoma.^[27]^ There have been several studies on the suppression of chondrosarcoma cells by targeting IDH mutations.^[28–30]^ Reducing matrix metalloproteinase (MMP) expression can also inhibit chondrosarcoma.^[24,31–33]^ Investigating the underlying mechanisms of chondrosarcoma pathogenesis and identifying effective therapeutic targets are urgent priorities.

Subtype study: the top 10 most frequent keywords “Mesenchymal Chondrosarcoma” and “Extraskeletal Myxoid Chondrosarcoma,” and keywords in the red cluster (Fig. 6) suggested that subtypes of chondrosarcoma, particularly the above mentioned 2 subtypes are also a research hotspot. Micaily et al suggested that the histological grading of chondrosarcoma is 1 of the most important factors that help identify prognosis.^[5]^ Eighty-five percent of chondrosarcomas are histologically classified as conventional chondrosarcomas and can be subdivided into central or peripheral types, while the other 15% include dedifferentiated, mesenchymal, clear cell, and myxoid. Most unconventional chondrosarcomas grow slowly and have limited metastatic potential. However, 5% to 10% of chondrosarcomas, including mesenchymal chondrosarcoma, have high metastatic potential and poor prognosis.^[34,35]^ Moreover, extraskeletal myxoid chondrosarcoma is an ultrarare subtype with unclear differentiation and no certain treatment modality for the time being.^[36,37]^ Both subtypes are refractory chondrosarcoma types with no effective therapeutic methods and a poor prognosis. These 2 subtypes are hot spots in the field of chondrosarcoma and deserve further exploration.

Combining the above 3 points, the next hotspot will probably be targeted therapy for refractory chondrosarcomas.

## 5. Conclusion

This study analyzed chondrosarcoma-related publications from 2000 to 2021. The number of publications in chondrosarcoma research field has increased in recent years. The most influential author is Tang Chih-Hsin (China Medical University). The 2 institutions that contributed the most were China Medical University and Leiden University. The number of publications from China has increased dramatically in recent years, whereas the United States remains the authority in the field of chondrosarcoma. Cancer is 1 of the most influential journals in this field. Furthermore, targeted therapy for refractory chondrosarcomas may be the next research hotspot.

## Author contributions

**Conceptualization:** Yusheng Li, Jun Zhang.

**Data curation:** Yuming Yao, Guang Yang, Bingzhou Ji.

**Formal analysis:** Guang Yang, Bingzhou Ji.

**Funding acquisition:** Guang Yang, Yusheng Li.

**Investigation:** Yuming Yao.

**Methodology:** Ruhao Zhou, Guang Yang, Bingzhou Ji.

**Validation:** Ruhao Zhou, Yusheng Li.

**Visualization:** Yuming Yao, Bingzhou Ji.

**Writing – original draft:** Yuming Yao, Guang Yang.

**Writing – review & editing:** Yusheng Li, Jun Zhang.
